# Embodied cognition of aging

**DOI:** 10.3389/fpsyg.2015.00463

**Published:** 2015-04-16

**Authors:** Guillaume T. Vallet

**Affiliations:** ^1^Centre de Recherche, Institut Universitaire de Gériatrie de MontréalMontréal, QC, Canada; ^2^Department of Psychology, Université de MontréalMontréal, QC, Canada

**Keywords:** embodiment, aging, dementia, sensory-motor processing, Alzheimer's disease, Parkinson disease

## Abstract

Embodiment is revolutionizing the way we consider cognition by incorporating the influence of our body and of the current context within cognitive processing. A growing number of studies which support this view of cognition in young adults stands in stark contrast with the lack of evidence in favor of this view in the field of normal aging and neurocognitive disorders. Nonetheless, the validation of embodiment assumptions on the whole spectrum of cognition is a mandatory step in order for embodied cognition theories to become theories of human cognition. More pragmatically, aging populations represent a perfect target to test embodied cognition theories due to concomitant changes in sensory, motor and cognitive functioning that occur in aging, since these theories predict direct interactions between them. Finally, the new perspectives on cognition provided by these theories might also open new research avenues and new clinical applications in the field of aging. The present article aims at showing the value and interest to explore embodiment in normal and abnormal aging as well as introducing some potential theoretical and clinical applications.

## 1. Introduction

In the last decades, human mind was thought to work as a computer, manipulating abstract symbols through different processing steps using clear rules (see Fodor, 1975). These rules are defined as insensitive to contextual variations, except when the context itself becomes an input of the cognitive system. A cat should always be a mammal with four legs regardless of the current situation (Collins, 1975). However, different places should trigger different personal memories (Tulving et al., 1983). This approach, known as cognitivism, was dominant in the study of cognition until recently when embodiment, also known as enactivism (Varela et al., 1991), started to revolutionize the way we conceive cognition (e.g., Glenberg et al., 2013).

In contrast to cognitivism, embodiment focuses on the interactions between cognition, the body and the environment. Cognitive processing is supposed to be directly impacted by the situation in which it occurs. A chair is processed differently when you are tired compared to when you want to change a light bulb (e.g., Barsalou, 1999; Pecher et al., 2003). Therefore, action as well as sensory and motor components are at the core of cognitive processes (Wilson, 2002). Embodiment has been successfully applied to numerous fields of cognition including language (Casasanto, 2011), memory (Versace et al., 2014), attention (Bradley, 2007), or action (Zmigrod and Hommel, 2013). Surprisingly, these theories remain almost unexplored in neuropsychology.

The present perspective aims at showing the value and interest to explore and apply embodied cognition theories to aging as an alternative to more traditional views. The first reason is theoretical. In order to become a theory of human cognition, embodiment should be validated across the whole spectrum of normal cognitive development from children to elderly adults. Embodied theories should furthermore be able to explain specific cognitive dysfunctions such as those occurring in neurocognitive disorders. A growing number of results are reported in babies and children with or without cognitive disorders (e.g., Wellsby and Pexman, 2014). Yet, only very few articles are published in the field of aging (e.g., Dijkstra et al., 2007), and even fewer articles tackle neurocognitive disorders from an embodied perspective (e.g., Vallet et al., 2013a). The second reason is more pragmatic. The specific changes occurring in aging make this population particularly interesting and relevant to investigate, including some of the core assumptions of embodiment presented below. Moreover, the inclusion of the body and the context in cognition should open new research avenues and may also lead to new clinical evaluation and remediation methods of cognitive functions. We will first present some key points about aging that will serve as the foundation for the next sections, which will deal with the application of embodiment to healthy aging and some neurocognitive disorders. We will focus on the interaction between cognition and (a) motor/action and (b) senses/perception based on theoretical arguments and when available, experimental evidence.

## 2. Normal aging and neurocognitive disorders

Becoming older is associated with changes in almost all spheres of the individual including physical condition, the senses, brain function, and cognition. Each sensory organ is affected in aging (Ulfhak et al., 2002), resulting in a decline in perception, including higher perceptual thresholds (Fozard and Gordon-Salant, 2001). Similar phenomena occurs in the motor system with a loss of motor neurons, decrease in the muscle mass (e.g., Vandervoort, 2002), decrease in the strength, resulting in gait and balance alterations (Boelens et al., 2013).

As the brain suffers from aging, cognition is also changing. Elderly adults show the greatest decline in cognitive speed of processing (Salthouse, 2000), attention, and executive functions (Greenwood, 2000) as well as in some aspects of episodic memory, mainly in free recall (Danckert and Craik, 2013). In contrast, most aspects of language, semantic memory and more automatic aspects of attention or memory remain preserved (Glisky, 2007).

Most neurocognitive disorders are also accompanied by sensory and perceptual impairments. These deficits, however, are more important in such disorders than those occurring in normal aging. For instance, the alteration of the nervous system in Alzheimer's disease (AD) causes significant decline in touch (Stephen et al., 2010), audition (Gates et al., 2011), vision (Kirby et al., 2010), and so forth. Yet, basic perception tasks such as detection of visual features (e.g., orientation, colors, etc.) and perceptual priming tasks remain preserved (Fleischman et al., 2005; Joubert et al., 2010). Motor and movement disorders are also very common in neurocognitive disorders, especially in Parkinson Disease (PD, Beitz, 2014) and Lewy body's dementia (Molano, 2013). Cognition is also more deeply affected than in normal aging with predominant episodic memory deficits in AD (Bäckman et al., 2005) and dysexecutive syndrome in PD (Dirnberger and Jahanshahi, 2013).

The concomitant changes of perception, action, and cognition in normal aging make this population particularly relevant to study embodied cognition theories. Indeed, these theories posit a causal relationship between sensory, motor functioning and cognition (Glenberg et al., 2013). As predicted, numerous studies have shown significant associations between altered senses/perception and cognition in aging (e.g., Lindenberger and Baltes, 1997; Madan and Singhal, 2012), but only a few have explored such a relation between motor/action and cognition (e.g., Lindenberger and Baltes, 1994; Verlinden et al., 2014). We will present the main interpretations of these relations and we will then focus on the possible implications within an embodied perspective. The cognitive impairment in neurocognitive disorders is much more important than that reported in healthy aging. In contrast, these populations differ much less in terms of their sensorimotor functioning. Therefore, the issue here is more about the ability of embodied cognition theories to explain cognitive impairment in neurocognitive disorders rather than the link between sensorimotor functioning and cognition as in normal aging. For the sake of clarity, we will only present data in PD and AD as they represent the most frequent dementia with motor and sensory impairment, respectively.

## 3. Action, motor components and cognition in aging

The relationship between motor/action and cognition in aging is mainly explained by physical health or by a common underlying cause namely the dopamine depletion. A better cardiovascular health directly improves cognition as brain function relies on a healthy vascular function (Debette et al., 2011). It is a well-known fact that motor activity, such as involved, in physical activity improves cognition at all ages (for a review, see Hillman et al., 2008), an effect which has also been found in Mild-Cognitive Impairment (MCI) and dementia (see Scherder et al., 2007, for review). The beneficial effects of sports on cognition are not limited to long-time practice of sports and are observed following new physical intervention programs in healthy aging (e.g., Colcombe and Kramer, 2003) as well as in MCI and AD (Heyn et al., 2004).

The impact on cognition may also just come from enhanced motor execution. Numerous neuropsychological tests involve motor execution so that faster execution and improved dexterity would lead to higher scores in these tests (e.g., video-gaming in aging van Muijden et al., 2012). Reversely, slower performance reported in aging appear to find its origin in motor processing rather than in sensory processing occurring in earlier steps (Yordanova et al., 2004).

Another way to account for these relationships is to posit a common underlying cause, such as the dopamine depletion hypothesis in aging. The aging brain produces less dopamine than the younger brain, which results in both motor and cognitive deficits such as attention and executive functions deficits (Volkow et al., 1998; Bäckman et al., 2005). This hypothesis could easily be applied to PD. Indeed, the degeneration of subcortical structures in PD causes dopamine depletion, which in turn causes motor and executive deficits (Dirnberger and Jahanshahi, 2013).

These hypotheses are not mutually exclusive and even if they are concordant with embodied cognition theories, they are not specific to them. More directly related to embodiment, motor execution and action are supposed to be necessary simulated in cognitive processes that rely or involve motor or action components. Therefore, better physical health and enhanced dexterity should also facilitate and even enhance action/motor simulation in cognitive processing and reversely. For instance, healthy elderly are less accurate than younger adults in the estimation of perceived weight judgment (Maguinness et al., 2013). This effect of aging can be partly explained in terms of motor simulation, in other words as a function of the real or perceived motor-efficacy of elderly adults (see Potter et al., 2009, for perceived efficacy). Loss of strength in aging, real or perceived, might lead to increase difficulties lifting objects leading therefore to an overestimation of their weight.

As predicted by embodiment, several studies found that the body itself influences cognition (Casasanto, 2011; Osiurak et al., 2014). One study found that our body posture influence the retrieval of autobiographical memories in both young and older adults (Dijkstra et al., 2007). This suggests that memories depend on the context of the body as predicted by the embodied cognition theories. Following the same logic, action/motor impairment reported in PD should not only impact how they physically interact with the environment, but should also change their cognitive representations and processing of the world (Poliakoff, 2013).

Yet, the most common findings in embodiment deal with language and how the comprehension of action related sentences require motor simulation (Zwaan and Yaxley, 2004). This embodiment of language in action has been reported in healthy elderly adults (Dijkstra et al., 2004), as well as in patients with AD (De Scalzi et al., 2014) since they rely on the necessary simulation of the motor components involved in language to understand it. It may also explain how motor impairments in PD are associated with altered language comprehension of literal and symbolic action related/based sentences (Fernandino et al., 2013).

Finally, it is worth noting that different studies have shown that motor simulation in word processing occurs in the same brain areas that are responsible for real planning and execution of actions (e.g., Hauk and Pulvermüller, 2004). The overlap argues in favor of common resources and perhaps of common units between motor, action, and perception (e.g., Hommel, 2004). The deterioration of motor and action representations in PD should result in the same degradation of mental simulation of whole-body images (Conson et al., 2014) and the ability to judge the weight to be lifted by another person (Poliakoff et al., 2010).

## 4. Perception, sensory components and cognition in aging

Since the sixties, a growing number of studies have reported an association between the alteration of senses and cognition in healthy aging (Schaie et al., 1964) to nowadays (Baldwin and Ash, 2011). These links are predicted by embodiment, but they generally are explained in terms of three main hypotheses. According to the cognitive load hypothesis (Baltes and Lindenberger, 1997), degraded perception leads to higher cognitive demand which cause in turn cognitive alterations for more demanding tasks or when fatigue occurs. For the sensory deprivation hypothesis (Valentijn et al., 2005), the decrease of sensory input results from the neuronal atrophy which in turn causes cognitive decline. This hypothesis appears congruent with embodiment as it is compatible with the assumption of common units for perception and cognition. This idea of brain atrophy is also part of the common cause theory which states that a third factor, such as a non specific alteration of the central nervous system, is responsible for the sensory and cognitive decline (Lindenberger and Baltes, 1997). Finally, one could argue that for motor performance and cognition, degraded perception should result in more time to complete the task, and consequently lead to poorer scores on cognitive tasks (Gussekloo et al., 2005). Nonetheless, this last hypothesis appears unlikely. Indeed, young and middle-aged adults under age-simulation conditions of reduced visual acuity, auditory acuity, or both did not exhibited lower cognitive performance relative to control conditions (Baltes et al., 2001; see also Scialfa, 2002).

Beyond these mutual influences of sensory/perceptual functioning on cognition, embodiment states that cognition is grounded in these perceptual components (Pecher and Zwaan, 2005). According to some authors, common resources and perhaps common units underlie perception and memory (e.g., Versace et al., 2014) as well as perception and action. Degraded perception should thus constitute degraded units in cognitive processing which in turn impaired cognitive performance. As a consequence, not only language should be grounded in sensorimotor components, but also conceptual knowledge (for a review, see Barsalou, 2008). Regarding sensory components, only two studies have explored this issue in aging (Vallet et al., 2011, 2013b) and found that healthy elderly adults exhibit grounded conceptual knowledge to the same extent as young adults.

The embodiment of conceptual knowledge could also be applied to AD. However, the disconnection syndrome that characterizes AD should impact the embodiment of conceptual knowledge in patients suffering from this disease. Some of the brain structures are disconnected from each other, mainly in the medial temporal region and posterior areas of the cortex (Delbeuck et al., 2003). Therefore, the necessary simulation of sensory components required to process a concept may not occur due to this neuroanatomical disconnection (Vallet et al., 2013a). In contrast to healthy young and elderly adults, AD patients did not exhibit perceptual cross-modal priming effects in an identification task. The lack of priming was surprising according to the classical approaches of cognition, since perceptual processing such as revealed by the priming effect is known to remain preserved even in moderate stages of the disease. In other words, embodied cognition theories have shed new light on an on-going debate.

Grounded knowledge also constitutes an interesting starting point to explore the changes in cognition due to aging with and without cognitive disorders. If knowledge remains grounded in its sensorimotor components, the degradation of these components should directly impact how knowledge is retrieved and used, i.e., memory performance. For instance, a recent study questioned the classical view of recognition impairment in healthy elderly adults by showing that they tend to perceive new items as old rather than old items as new (Yeung et al., 2013). This effect was found for high-interference items, items with a significant degree of feature overlap. According to the authors, it is the degradation of the perirhinal cortex which is known to be involved in higher-level visual processing that cause this effect. In other words, memory traces of healthy seniors may be slightly degraded or perhaps slightly less distinctive. The role of distinctive processing was put forward to explain the increase of false memories in aging (Butler et al., 2010).

Furthermore, this trace distinctiveness is thought to be at the core of the emergence of episodic memory when associated with integration according to some embodied cognition theories (see Brunel et al., 2013; Versace et al., 2014). Given that memory emergence relies on the simulation/enactment of the different components of the memory trace, episodic memory should then require the dynamic integration of these different simulations into one coherent representation (see Versace et al., 2014). Yet, it is now established that integration is impaired in AD in multisensory tasks (Festa et al., 2005), working memory tasks (Parra et al., 2009), and episodic memory tasks (Stoub et al., 2006), resulting from the disconnection syndrome. Therefore, episodic memory impairment in AD may result, at least partially, from the integration deficit reported in AD (see Delbeuck et al., 2007).

The distinctiveness and the role of (re)-integration in memory suggest that multisensory stimulation should enhance memory and possibly cognition. It was observed that programs based on Multi-Sensory Stimulation (MSS), also called snoezelen, increase the well-being of patients with dementia in health-care houses. Even if some reviews did not find any specific effectiveness of these programs (Chung and Lai, 2002), recent data support beneficial effects on mood, behavior, communication (for review see Sánchez et al., 2013), and sometimes on cognition as assessed by MMSE (Ozdemir and Akdemir, 2009; Maseda et al., 2014). This possible effect on cognition is predicted by embodiment, but not by cognitivism. A future direction to improve stimulation/remediation programs may then be to enhance the distinctiveness of memory traces, along with effort develop the integration of the different components of the traces.

## 5. Conclusions

Numerous studies have reported the mutual influence of motor/action and sensory/perception functions on the one hand and cognition on the other hand. These relations fit naturally within embodied cognition theories since embodiment assumes cognition is in interaction with the body and the environment. Possibly due to sensory-motor and cognition decline occurring in aging, these associations appear even stronger in this population (Baltes and Lindenberger, 1997). Yet, embodiment remains marginally explored and applied to aging.

Nonetheless, the few studies published to date seem to have successfully validated embodied assumptions in normal aging and in neurocognitive disorders. The main result is that healthy elderly adults exhibit grounded conceptual knowledge and language in perceptual features (Vallet et al., 2013b) and motor features (Dijkstra et al., 2004). This seems to highlight one of the key differences between embodiment and cognitivism on the whole spectrum of normal cognition, ranging from children (Wellsby and Pexman, 2014) to elderly (Vallet et al., 2013b). The particular profile of healthy older adults viewed in an embodied perspective also contributes to formulate new hypotheses about their performance. As briefly mentioned above, the effect of age on the perihinal cortex may cause greater confusion to distinguish one memory trace from another (e.g., Butler et al., 2010; Yeung et al., 2013).

The same principles may be applied to neurocognitive disorders, with validation coming from grounded language in PD (Cardona et al., 2014) and AD (De Scalzi et al., 2014). The respective motor impairment in PD and in cognition in AD also represent new avenues of research in embodiment (e.g., Poliakoff et al., 2010; Vallet et al., 2013a). One of the most exciting perspective however remains the possible development of new interventions based on radically different assumptions than those used within a cognitivist perspective to help patients. One can imagine to focus on the dynamic nature of memory to improve episodic memory, or to focus on the whole body functioning of an individual to improve cognition. For instance, a recent study claims to revert cognitive decline in dementia following a drastic change of life-style without any cognitive stimulation program or dementia related drugs (Bredesen, 2014). This program is based on healthy diet, good sleep habits, meditation and exercises combined with medication used to maximized physiological changes (hormone balance, mitochondrial function, etc.).

Despite the lack of research in these areas, normal aging, as well as neurocognitive disorders, appear as particularly interesting and useful targets to explore and apply embodiment. We hope that this brief presentation will encourage cognitivists and neuropsychologists to further explore and consider these new avenues of research.

### Conflict of interest statement

The authors declare that the research was conducted in the absence of any commercial or financial relationships that could be construed as a potential conflict of interest.
